# National and sub-national under-five mortality profiles in Peru: a basis for informed policy decisions

**DOI:** 10.1186/1471-2458-6-173

**Published:** 2006-07-04

**Authors:** Luis Huicho, Miguel Trelles, Fernando Gonzales

**Affiliations:** 1 Department of Paediatrics, School of Medicine, Universidad Nacional Mayor de San Marcos and Instituto de Salud del Niño, LI 05, Lima, Peru; 2 Department of Paediatrics, School of Medicine, Universidad Peruana Cayetano Heredia, LI 05, Lima, Peru; 3 Pan American Health Organization, LI 05, Lima, Peru

## Abstract

**Background:**

Information on profiles for under-five causes of death is important to guide choice of child-survival interventions. Global level data have been published, but information at country level is scarce. We aimed at defining national and departmental trends and profiles of under-five mortality in Peru from 1996 through 2000.

**Methods:**

We used the Ministry of Health registered under-five mortality data. For correction of under-registration, a model life-table that fitted the age distribution of the population and of registered deaths was identified for each year. The mortality rates corresponding to these model life-tables were then assigned to each department in each particular year. Cumulative reduction in under-five mortality rate in the 1996–2000 period was estimated calculating the annual reduction slope for each department. Departmental level mortality profiles were constructed. Differences in mortality profiles and in mortality reduction between coastal, andean and jungle regions were also assessed.

**Results:**

At country level, only 4 causes (pneumonia, diarrhoea, neonatal diseases and injuries) accounted for 68% of all deaths in 1996, and for 62% in 2000. There was 32.7% of under-five death reduction from 1996 to 2000. Diarrhoea and pneumonia deaths decreased by 84.5% and 41.8%, respectively, mainly in the andean region, whereas deaths due to neonatal causes and injuries decreased by 37.2% and 21.7%. For 1996–2000 period, the andean, coast and jungle regions accounted for 52.4%, 33.1% and 14.4% of deaths, respectively. These regions represent 41.0%, 46.4% and 12.6% of under-five population. Both diarrhoea and pneumonia constitute 30.6% of under-five deaths in the andean region. As a proportion, neonatal deaths remained stable in the country from 1996 to 2000, accounting for about 30% of under-five deaths, whereas injuries and "other" causes, including congenital anomalies, increased by about 5%.

**Conclusion:**

Under-five mortality declined substantially in all departments from 1996 to 2000, which is explained mostly by reduction in diarrhoea and pneumonia deaths, particularly in the andean region. There is the need to emphasize interventions to reduce neonatal deaths and emerging causes of death such as injuries and congenital anomalies.

## Background

Patterns of under-five mortality are heterogeneous among different regions in the world and within countries. Data on global causes of child death have been published. They show that 90% of deaths occur in 42 countries, diarrhoea, pneumonia and neonatal disorders explaining about 74% of these deaths [1,2]. This information has been built mostly on the basis of study data or national demographic and health survey (DHS) information, particularly for low-income countries, where vital registration systems are incomplete and/or unreliable [1,2]. It is noteworthy that the completeness and quality of information is lowest in the poorest countries that are in most need of child-survival interventions [2].

Universal and equitable implementation of cost-effective child survival interventions building on local epidemiology through outreach, family-community care, and facility-based clinical care has been proposed for achieving the reduction of under-five mortality rates by two thirds to year 2015 [3-5]. Although global level data on causes of death of children have been published, information at country and particularly at local level is scarce. Most developing countries have no comprehensive strategy for health information managements. In many of them vital event monitoring systems function poorly. In South Asia, only 60 percent of births are registered, and in Africa, only 30 percent [6]. In many countries of the Americas, the coverage of the civil registration system is incomplete, and in some countries the population covered by available mortality data needs to be further clarified [7]. Within countries, the completeness of registration is known to vary according to geographic area and age group [7]. Under registration of deaths is low in Costa Rica (4%) and intermediate in the Bahamas, Brazil, Guyana, Mexico, Panama, and Suriname, which have estimated under registration ranging between 11% and 19%, and appear to be on the way to achieving satisfactory levels of death registration [7]. Another 9 countries continue to have serious under registration problems, with estimates ranging between 20% and 92 [7]. No data from civil registration sources are available for Bolivia [7]. Under registration is greater for infant deaths than for deaths occurring at older ages. There are efforts to improve the health information systems in the developing countries. Alternatives to universal registration include the sample registration systems used in China and India and the demographic surveillance experiences in Tanzania [6]. The United Nations Statistics Division has developed guidelines for vital statistics systems to support countries in their development. The Pan American Heal organization (PAHO) has launched the Regional Core Health Data Initiative to allow a rapid access to basic information on the health situation in the countries of the region and it is also aimed at expanding the system to sub-national districts [8].

Peru is a lower-middle-income country divided politically in 24 departments and has three different regions, namely coast, andean and jungle. Each region has distinct epidemiological, socioeconomic and geographical characteristics. Human development index, percentage of families living in poverty, gross national product, other socioeconomic factors and child health indicators are comparatively better in Peru than those in most low-income countries [9]. However, these national figures hide great disparities among departments and even within them. A systematic information on Peruvian causes of under-five deaths at national and sub-national level is lacking. This information is important for making an informed choice for prioritizing child survival interventions. The total number of registered deaths in under-five children in Peru was estimated in 30,876 for 2000, which corresponds to 47.1 per 1,000 live births. This accounts for 18.9% of total deaths for all ages [10]. However, the proportion of under-registration of deaths in Peru is high (48.3%) and ranges from 1.5% in Callao (a coastal city close to Lima, the capital city of Peru) to 78.2% in Loreto (a jungle inner city) [7]. The high levels of under-registration are explained in part by the low priority level of the health information system. The health authorities are responsible for the recollection, distribution and quality assurance of the death certificates, all of which are still to be improved substantially. In addition, the accessibility to health services is still low in some remote areas of the country, particularly in rural settings.

Different under-five mortality rates and causes of death among departments and within them create the need to define regional profiles for an adequate planning of health policies and interventions. Thus we were prompted to outline the national and sub-national level under-five mortality in Peru. We aimed at determining the national and departmental trends and profiles of under-five mortality during the period 1996 through 2000, taking advantage of registered vital statistics and of methods for correcting under-registration.

## Methods

We used the registered child mortality data provided by the Peruvian Ministry of Health (MOH) for the period 1996 through 2000. These data were corrected for under-registration through a method developed by the Centro Latino-Americano de Demografia (CELADE) and adopted by PAHO [7]. This method is described in detail in the CELADE technical document [7]. The estimated under registration of deaths are based on a comparison of the crude death rates obtained using registered mortality provided by the MOH for the year period indicated, and the death rates estimated by using life table central death rates. The registered mortality data is tabulated for causes of death, age groups, and sex, estimates of the central death rates for the corresponding age groups and sex, obtained from life tables for 20 Latin American countries prepared and published by CELADE; and corresponding annual population estimates by age groups and sex. The registered mortality data is first adjusted for deaths unknown by age and sex. The number of deaths unknown by age are redistributed into known age groups by multiplying the number of deaths for each sex and age group by an adjustment factor, fa = D/Da, where D is the total number of deaths and Da is the number of deaths stated by age. A similar adjustment factor was used to redistribute the number of deaths in each age group not stated by sex. The rate calculations make some basic assumptions about the registered mortality data. First, All registered deaths coded to an external cause were in fact due to an external cause, and none of the registered deaths coded to other cause categories, including symptoms, signs, and ill-defined conditions (SSI), were really due to external causes. All deaths assigned to SSI can thus be proportionately redistributed among other non-external cause categories, age groups, and sex, under the assumption that the SSI deaths follow the same distribution as that observed among registered deaths from non-external "defined" causes. Second, an estimate of the total number of deaths that actually occurred in a given year or time period was obtained by applying the corresponding quinquennial central death rates for each age and sex group from the life table to the population estimates and totaling the number of deaths in each age group by sex. By subtracting the number of registered deaths, an estimate of the number of unregistered deaths was obtained. The CELADE method also assumes that the distribution of unregistered deaths into cause categories, by age group and sex, is the same as that among registered deaths. Accordingly, unregistered deaths, including unregistered deaths due to external causes, were redistributed into corresponding cause categories by age and sex in the same proportions as the registered deaths. Third, estimated age and sex specific rates were calculated by accumulating all the registered and unregistered deaths in a given year or time period, by cause category and dividing by the sum of the corresponding estimated populations. The infant mortality rate was calculated using the estimated population under 1 year of age in the denominator, as the estimated number of live births was not available.

The results were compiled by PAHO and expressed as deaths per 100,000 children under-five. The Peruvian under-five mortality rate was multiplied by 4.7 and divided by 100, for obtaining figures comparable with deaths rates by 1,000 live births [11]. This calculation was made due to the fact that there are about 4.7 more under-five children than live births, according to current levels of mortality in Peru [11]. Cumulative reduction in under-five mortality rate in the 1996–2000 period was estimated calculating the annual reduction slope for each department, through fitting of a regression line of mortality rate levels on calendar years; the five-year cumulative reduction was obtained from the annual slopes. Differences in mortality patterns and in mortality reduction between coastal, andean and jungle regions were also assessed.

Our registered mortality data were previously validated by checking the correlations with well defined determinants of under-five mortality such as socioeconomic and environmental factors [11]. All these correlations were significant and in the expected direction, that is, departments with better socioeconomic and environmental conditions had lower under five mortality rates [11]. In addition, we also tried to validate the official registered statistics on under five mortality correlating them with the indirect under five mortality estimates from the national demographic and health surveys (DHS), provided on a departmental basis [11]. Although DHS-based estimates are affected by sampling variability and refer to a date about five years prior to the survey, they are not affected by under-registration since they rely on maternal report. The correlation between the registered under five mortality rates in 1996–2000 and indirect under five mortality rate estimates from the 2000 DHS was equal to 0.90 and highly significant (p < 0.001) [11].

For determining the departments according to different profiles of death, we grouped the causes of death following the WHO method reported in two background papers [1,2], that is, setting convenience thresholds for the different causes of death. The background papers used as reference for assigning the causes of death used several sources of data (Censuses, DHS, UN estimates, vital registration data, epidemiological data from studies, verbal autopsies, WHO programme estimates, and independent studies). All those sources allow the construction of age-specific mortality rates, life tables, cause-specific mortality rates, and of country level age-specific, sex-specific, and cause specific mortality estimates [2]. The predictions of the cause-of-death model used were validated using vital registration-based data from Mexico, that has a vital registration system that is considered complete, with 95% or more coverage of the system, so as there is unlikely to be any substantial selection bias in the proportional distribution of mortality by cause in children younger than 5 years in the vital registration data [1]. In our paper we set the cut-offs just above the national average proportion of death for each cause in 1996. We used 1996 as the reference year, allowing in this way the detection of the variations in the causes of death from 1996 through 2000.

## Results

**Figure 1:**
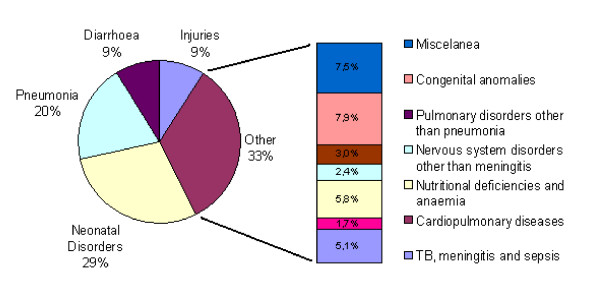
Causes of under-five mortality at country level for the period 1996–2000.

**Figure 2:**
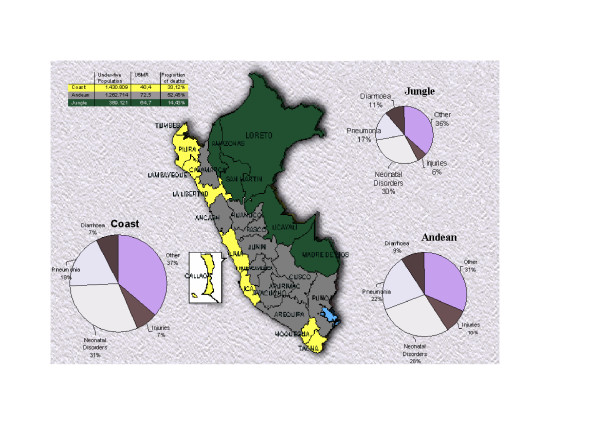
Geographic distribution of under-five mortality rate (U5MR), average for 1996–2000 period. Size of pies is proportionate to under-five population.

At country level there were 43,779 registered under five deaths in 1996, and 30,874 in 2000, which corresponds to 32.7% of reduction. The total number of under-five deaths for the period 1996–2000 was 186,178. Figure 1 depicts proportion of deaths by cause. Neonatal causes, pneumonia, and diarrhoea explain 29%, 20%, and 9% of all under-five deaths, respectively. For this period, when considered by region, the coast accounted for 61,540 deaths (33.1% of total deaths), the andean region for 97,442 (52.4%), and the jungle for 26,801 (14.4%) (Figure 2). Coastal and andean departments account for more than 85% of total deaths. The andean region alone accounts for more than half of all under-five deaths. Both pneumonia and diarrhoea constitute a greater proportion of deaths (31%) than neonatal causes (28%) in the andean region, in contrast to coast, where neonatal deaths predominate (31%) (Figure 2).

**Figure 3:**
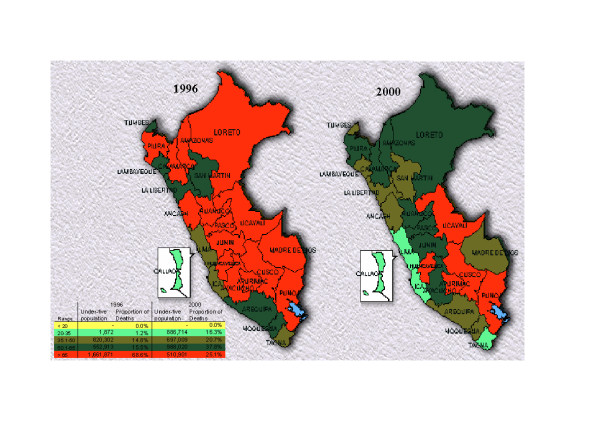
Variation of under-five mortality rate (U5MR) in Peruvian departments, 1996 through 2000.

Under-five mortality declined substantially in all departments from 1996 to 2000. Figure 3 shows departments categorized by range of under-five mortality rate. In 1996, those departments with more than 65 per 1,000 live births accounted for 68.6% of total under-five mortality. In 2000, this group explained 25.1% of overall under-five mortality. In 2000, the highest mortality rate for any single department was 76.8 per 1,000 live births (Puno), whereas in 1996 there were 9 departments with more than this mortality rate. Tables 1 and 2 show slope of mortality variation by cause, by geographical regions and by departments. Diarrhoea, pneumonia, injuries and neonatal deaths declined, particularly the first two, by 82.1%, 45.5%, 32.1% and 30.8%, respectively. All causes of death decreased in all regions, except injuries-related deaths that increased, but only in the andean region. Likewise, diarrhoea and pneumonia decreased in all departments, except in Loreto, where pneumonia increased, and in Tumbes, where diarrhoea also increased slightly (Table 2).

**Figure 4:**
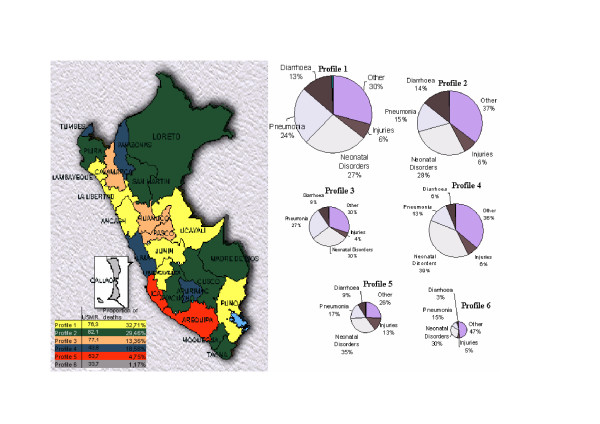
Under-five mortality rate (U5MR) for 1996 and profiles of departments. Size of pies is proportionate to under-five population.

**Figure 5:**
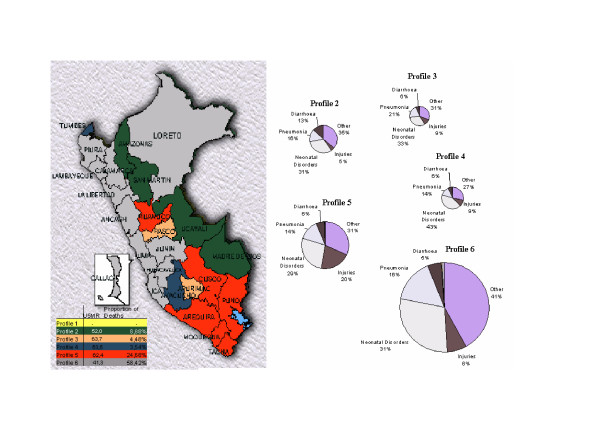
Under-five mortality rate (U5MR) for 2000 and profiles of departments. Size of pies is proportionate to under-five population.

Figures 4 and 5 and display profiles of under-five deaths for 1996 and 2000, respectively. Description of profiles and number of departments for each profile is shown in Table 3. In 1996, profile 1 and 2 departments accounted for 32.7% and 29.5% under-five deaths, respectively. The number of departments with profile 5 and 6 increased from 1996 to 2000. In 2000, profile 1 departments disappeared and profile 2 departments accounted only for 8.8% of total under-five deaths. The greatest increase occurred in profile 6, which varied from 1.17% to 58.4% of total deaths.

**Figure 6:**
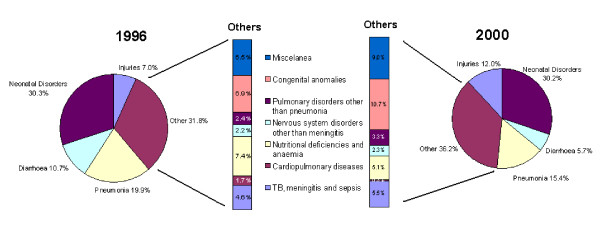
Causes of under-five mortality at country level for 1996 and 2000.

Figure 6 shows proportion of under-five mortality by cause for 1996 and 2000. As proportion of total under-five deaths, pneumonia and diarrhoea deaths decreased from 10.7% to 5.7% and from 19.9% to 15.4%, respectively. Injuries increased from 7.0% to 12.0%. Neonatal deaths remained stable from 1996 to 2000, accounting for about a third of total under-five deaths. In "other" category, congenital anomalies increased from 6.5% to 10.7% and nutritional deficiencies including anaemia decreased from 7.4% to 5.4%.

## Discussion

Under-five mortality rates (U5MR) decreased substantially by one third at country level in the period 1996 through 2000. Most causes of death decreased. Diarrhoea, pneumonia, injuries and neonatal deaths declined substantially, particularly the first two conditions. However, as a proportion of total under-five deaths, injuries and "other" increased by about 5%, whereas neonatal causes remained practically unchanged at national level, accounting for about one third of total under-five deaths. Also as a proportion of under-five deaths, congenital anomalies constitute an increasingly important cause of under-five deaths in Peru. At regional level, all causes of death also declined substantially in coast, andean and jungle regions, except for injuries, that increased in the andean region by more than a quarter. It is noteworthy that the lowest overall mortality declination and that due to diarrhoeal diseases occurred in the jungle.

In 1996, profiles 1, 2 and 3 accounted for more than three quarters of under-five deaths at national level, whereas in 2000 they had decreased dramatically and only explained 13.4% of these deaths. Seventeen departments (with 64% of total under-five population) showed these profiles in 1996, and only five (with 11% of national under-five population) showed these profiles in 2000. In fact, there was not any single department with profile 1 in 2000. This reveals, in most departments of Peru, a dramatic decline in diarrhoea and pneumonia deaths during this period and a proportionately higher importance of deaths due to neonatal causes, injuries and "other" causes of death which belong to profiles 4,5 and 6. This time-trend in mortality profiles shows a substantial improvement in those under-five causes of death that are more amenable to a change through child-survival interventions such as Integrated Management of Childhood Illness (IMCI) that address mainly deaths due to pneumonia, diarrhoea and malnutrition [12].

The results of this study reveal that the potential of child-survival interventions for Peru as a country and for their different regions and departments varied substantially during the study period. Consequently, interventions targeted exclusively to reduction of deaths due to pneumonia and diarrhoea irrespectively of the local epidemiology may have less impact than in the past, although they remain important for those regions where they are still leading causes of under-five deaths. By contrast, intervention packages addressing neonatal deaths, injuries and congenital malformations seem increasingly important now. In such a variable within-country context, caution must be exercised when trying to apply unique national child-survival strategies, which may not be applicable with the same strength to different regions of the country. The need to build on local epidemiology for identifying the most cost-effective packages of child interventions has been emphasized in several publications [3-5]. An additional challenge is the achievement of universal and equitable coverage for those interventions [13,14], through outreach, family-community care, and facility-based clinical care [15-17], and taking into account the importance of strengthening health systems [18,19]. Failing to include such considerations during the health policy decision making process will reduce substantially the potential impact of child health interventions [5]. We addressed here the need to define the local epidemiology of under-five causes of death.

Our study took advantage of registered mortality data corrected for under-registration, instead of indirect mortality information or study data, more frequently used when assessing the causes of death at global level [2]. The DHS surveys provide indirect estimates of child mortality based on birth histories, but estimates for the departmental-level are based on births that occurred in the 10 years before the survey, and thus the results from the Peruvian 2000 DHS (ENDES 2000) refer approximately to about 1995 [20]. However, the high levels of under-registration and the variable quality of routine registration of mortality data by cause of death from the Ministry of Health remains a problem. Such data may grossly underestimate and modify the levels, cause-specific structure and age-pattern of mortality, and it is also possible that some causes of death may be more or less reported than other causes. Without a gold standard against which one can compare the cause of death structure reported here, it is hard to determine with high level of certainty what real differences in levels and trends are over time in under-five mortality across the 24 departments of Peru from erratic differences. The CELADE method for correction of under-registration used in this report reduces such limitations, but it can not replace the urgent need of improving the health information system in Peru and in other developing countries, emphasizing on the vital registration system.

We also acknowledge that varying the convenience thresholds used for defining the different profiles of death may lead to the inclusion or exclusion of some departments from a due profile. However, these changes are not substantial and they still allow the identification of departments by the leading causes of death. Furthermore, we did not address the issue of multiple causes of death, which often act synergistically to produce under five mortality [1,21]. Undernutrition is also an important underlying cause of the majority of under five children deaths and thus it should be considered when determining the causes of death [1]. Health policymaking building on the basis of national and sub-national patterns of deaths in children should take these issues into consideration.

We also acknowledge that more disaggregated mortality data would reveal further disparities in under-five profiles of death within departments. Unfortunately, the quality and completeness of health information decrease dramatically when trying to get mortality data for geographical or political divisions smaller than the departmental level. Support of health information systems at local level has been included as part of the efforts to strengthen health systems [22], and hopefully this support will help to the improvement of vital registration systems.

## Conclusion

In conclusion, under-five mortality in Peru declined substantially in all departments from 1996 to 2000, which is explained mostly by reduction in diarrhoea and pneumonia deaths, particularly in the andean region. Thus there is the need to emphasize on the implementation of interventions aimed at reducing newborn causes of death and emerging causes such as injuries and congenital anomalies.

## Competing interests

The author(s) declare that they have no competing interests.

## Authors' contributions

LH conceived the study. LH, MT and FG took part in design, data collection, management and analysis. All authors contributed to interpretation of the data and writing of the report. All authors read and approved the final manuscript.

## Pre-publication history

The pre-publication history for this paper can be accessed here:



## Figures and Tables

**Table 1:** Percentage of under-five mortality variation from 1996–2000 by regions, Peru.

	**Other**	**Injuries**	**Neonatal Disorders**	**Pneumonia**	**Diarrhoea**	**All causes**
**Coast**	-15,4%	-32,1%	-30,8%	-45,5%	-82,1%	-32,8%
**Andean**	-23,5%	25,7%	-27,8%	-49,7%	-97,5%	-33,4%
**Jungle**	-27,0%	-24,3%	-33,0%	-17,9%	-49,2%	-30,5%
**Country level**	-15,4%	-32,1%	-30,8%	-45,5%	-82,1%	-32,8%

**Table 2:** Slope of registered under-five mortality rate from 1996 to 2000 by departments.

	**Region**	**Other (%)**	**Injuries (%)**	**Neonatal (%)**	**Pneumonia (%)**	**Diarrhoea (%)**	**All causes (%)**	**Under five population**
**PERU**		-23.04	21.67	-37.20	-41.85	-84.54	-32.72	3,106,958
**Amazonas**	3	-30.49	-71.09	-30.14	-10.63	-51.63	-34.23	62,612
**Ancash**	2	-31.73	29.62	-26.96	-45.05	-53.29	-31.09	133,922
**Apurimac**	2	-25.31	-12.49	-44.26	-9.98	-106.97	-34.43	68,402
**Arequipa**	2	-5.24	30.57	-36.42	-23.14	-108.54	-20.67	109,216
**Ayacucho**	2	-48.50	-24.74	1.63	-86.57	-138.24	-41.42	76,611
**Cajamarca**	2	6.56	28.85	-22.97	-70.01	-108.42	-28.47	208,414
**Callao**	1	-36.71	-9.57	-36.89	-17.29	-51.86	-32.53	71,872
**Cusco**	2	-46.60	46.52	-42.66	-58.12	-116.90	-41.69	161,287
**Huancavelica**	2	-8.05	23.73	-33.84	-74.61	-99.71	-41.58	66,897
**Huanuco**	2	-36.44	46.72	-18.61	-50.39	-52.69	-29.63	113,225
**Ica**	1	-3.24	-49.64	-36.06	-40.00	-77.81	-31.39	73,054
**Junín**	2	7.38	3.06	-23.84	-62.82	-76.53	-29.92	156,244
**La Libertad**	1	8.29	-3.87	-59.49	-25.24	-83.39	-26.31	175,951
**Lambayeque**	1	-46.48	-15.21	-10.75	-34.68	-107.79	-39.37	130,479
**Lima**	1	-15.34	-25.53	-47.03	-7.94	-91.24	-28.81	719,506
**Loreto**	3	-29.02	-11.07	-28.49	49.12	-83.81	-28.76	138,306
**Madre de Dios**	3	-40.61	-9.41	-30.06	-57.30	-8.14	-32.22	12,856
**Moquegua**	1	37.57	-68.68	-13.39	-50.34	-122.22	-26.13	14,349
**Pasco**	2	-20.66	28.69	-28.97	-22.59	-167.41	-30.01	35,443
**Piura**	1	-21.20	-28.39	-33.92	-44.85	-61.89	-34.46	207,653
**Puno**	2	-25.20	46.41	-31.35	-43.82	-71.40	-29.56	157,923
**San Martín**	3	-17.59	-67.53	-41.33	-49.56	-73.98	-40.91	100,633
**Tacna**	1	-56.11	21.24	-16.43	-78.92	-129.46	-48.48	27,742
**Tumbes**	1	-7.08	-42.54	-25.05	-113.16	1.20	-28.99	22,285
**Ucayali**	3	-18.25	28.30	-37.04	-9.68	-39.45	-20.69	62,076

1, Coast; 2, Andean; 3, Jungle

**Table 3:** Profiles of death and number of departments for each profile, 1996 and 2000.

**Profile**	**Description**	**Number of departments**	
		**1996**	**2000**
**1**	Diarrhoea >10% and Pneumonia > 20%	7	0
**2**	Diarrhoea >10%	8	4
**3**	Pneumonia >20%	3	2
**4**	Neonatal deaths >35%, Diarrhoea <10% and Pneumonia <20%	4	4
**5**	Injuries >10%, Diarrhoea <10%, Pneumonia <20% and Neonatal deaths <35%	2	6
**6**	Injuries <10%, Diarrhoea <10%, Pneumonia <20% and Neonatal deaths <35%	1	9
